# Comparative Study on Functional Effects of Allotransplantation of Bone Marrow Stromal Cells and Adipose Derived Stromal Vascular Fraction on Tendon Repair: A Biomechanical Study in Rabbits 

**Published:** 2014-10-04

**Authors:** Mehdi Behfar, Sara Javanmardi, Farshid Sarrafzadeh-Rezaei

**Affiliations:** 1 Department of Clinical Sciences, Faculty of Veterinary Medicine, Urmia University, Urmia, Iran; 2 Department of Clinical Sciences, Faculty of Veterinary Medicine, University of Tabriz, Tabriz, Iran

**Keywords:** Tendon, Tensile Strength, Adipose Tissue, Bone Marrow, Transplantation

## Abstract

**Objective:**

Tendon never returns to its complete biological and mechanical properties after repair. Bone marrow and, recently, adipose tissue have been used as sources of mesenchymal stem cells which have been proven to enhance tendon healing. In the present study, we compared the effects of allotransplantation of bone marrow derived mesenchymal stromal cells (BMSCs) and adipose derived stromal vascular fraction (SVF) on tendon mechanical properties after experimentally induced flexor tendon transection.

**Materials and Methods:**

In this experimental study, we used 48 adult male New Zealand white rabbits. Twelve of rabbits were used as donors of bone marrow and adipose tissue, the rest were divided into control and treatment groups. The injury model was a unilateral complete transection of the deep digital flexor tendon. Immediately after suture repair, 4×10^6^cells of either fresh SVF from enzymatic digestion of adipose tissue or cultured BMSCs were intratendinously injected into tendon stumps in the treatment groups. Controls received phosphate-buffered saline (PBS). Immobilization with a cast was continued for two weeks after surgery. Animals were sacrificed three and eight weeks after surgery and tendons underwent mechanical evaluations. The differences among the groups were analyzed using the analysis of variance (ANOVA) test followed by Tukey’s multiple comparisons test.

**Results:**

Stromal cell transplantation resulted in a significant increase in ultimate and yield loads, energy absorption, and stress of repairs compared to the controls. However, there were no statistically significant changes detected in terms of stiffness. In comparison, we observed no significant differences at the third week between SVF and BMSCs treated tendons in terms of all load related properties. However, at the eighth week SVF transplantation resulted in significantly increased energy absorption, stress and stiffness compared to BMSCs.

**Conclusion:**

The enhanced biomechanical properties of repairs in this study advocates the application of adipose derived SVF as an excellent source of multipotent cells instead of traditional BMSCs and may seem more encouraging in cell-based therapy for tendon injuries.

## Introduction

Severe tendon injuries are difficult to manage and surgically repaired tendons do not fully restore function (1). Due to the low cellularity and low mitotic activity of the tendon (2), its injuries are slow to heal and healed tendons rarely regain their original strength and elasticity (3). The inferior healing causes prolonged recovery times and a high rate of re-injury (4). An advanced procedure for treating tendon injuries includes injecting stromal cells into the injured areas to support healing of the tissue (5-8). Stromal cells have great potential in improving the biologic healing process since they deliver a self-renewing population of multipotent cells (9). Stromal cell therapy in animal models has been utilized in treatment of tendon injuries with the initial source of cells derived from bone marrow (10). Recently, adipose tissue has been described as a rich source of stromal cells (11). It has been reported that these cells are multipotent cells which can differentiate into tendon cells and may accelerate tendon regeneration and repair (12). Although the *in vitro* properties of mesenchymal cells from bone marrow and adipose tissue have been compared before (13-15), there is no report comparing functional effects of these cells on tendon healing. Here, we represent the first report of mechanical properties of tendon repairs in response to transplantation of bone marrow derived mesenchymal stromal cells (BMSCs) and uncultured adipose derived stromal cells, known as stromal vascular fraction (SVF), in an experimentally induced tendon transection model in rabbits.

## Materials and Methods

### Animals

In this experimental study, we used 48 adult male New Zealand white rabbits that weighed 2.5-3.0 kg. During the study animals were housed individually in stainless steel cages (60×55×40 cm) under standard conditions and given food (commercial rabbit pellet) and water ad libitum. A group of 12 rabbits were used as donors of bone marrow and adipose tissue, whereas the remainder of the rabbits were divided randomly and equally into control and treatment groups of 12 animals per group.

### Isolation and expansion of bone marrow derived mesenchymal stem cells (BMSCs)

**Fig 1:**
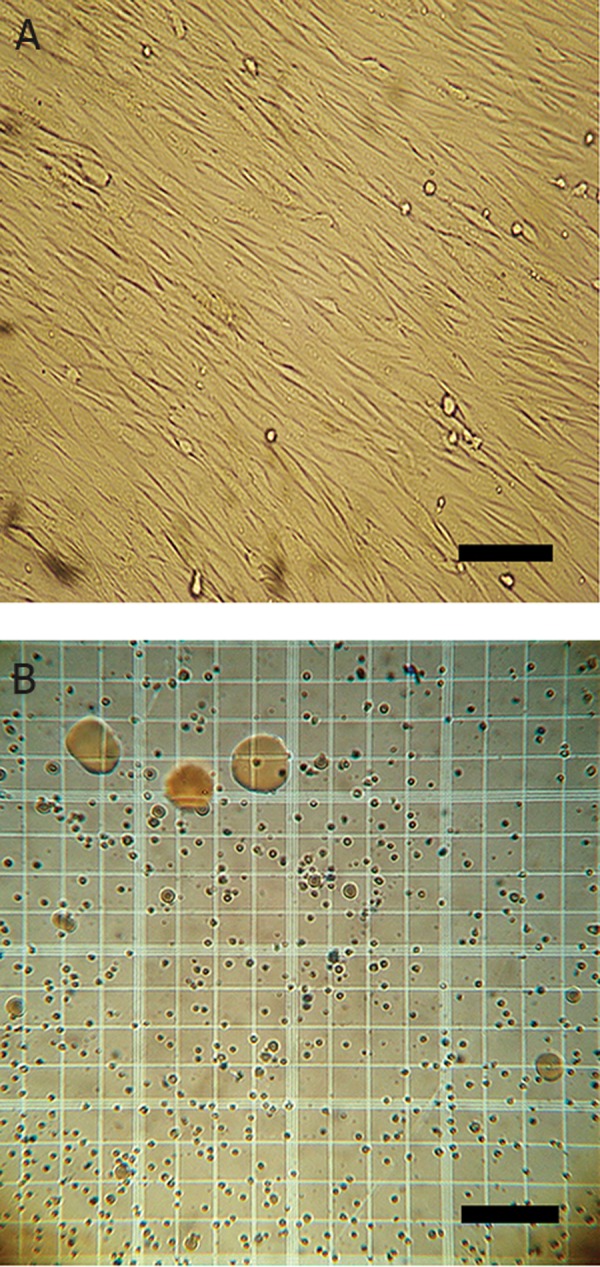
A. Spindle-shaped fibroblast-like mesenchymal stromal cells isolated from rabbit bone marrow. B. Nucleated cells in stromal vascular fraction isolated from rabbit adipose tissue (scale bar =100 μm).

Donor rabbits were anaesthetized by intramuscular injection of xylazine HCl (5 mg/kg, Alfasan, The Netherlands) and ketamine HCl (40 mg/kg, Alfasan, The Netherlands). According to previous studies (16, 17), bone marrow was aseptically aspirated from the iliac crest and collected into polypropylene tubes that contained 1000 unit/ mL preservative-free heparin. The bone marrow and heparin were mixed. Nucleated cells were isolated by density gradient centrifugation over Ficoll/pague (Pharmacia).The nucleated cell layers were carefully removed and resuspended in a culture medium that contained Dulbecco’s modified eagle’s medium (DMEM, Sigma Co., St. Louis, MO, USA), 15% fetal bovine serum (FBS, Gibco, Carlsbad, CA, USA), 100 unit/mL penicillin (Sigma-Aldrich, USA) and 100 μg/mL streptomycin (Sigma-Aldrich, USA). The nucleated cells were plated at a density of 5×10^6^ nucleated cells in T-75 flasks and grown at a temperature of 37˚C and 5% CO_2_ in a humidified tissue-culture incubator. After five days, the contents of the flask were removed and washed with medium. We discarded the non-adherent cells and cultured the adherent cells. The medium was changed every three days. After about 14 days, cells were trypsinized (0.25% trypsin/EDTA, Gibco, Grandisland, USA) at 70- 80% confluency and then serially subcultured. Cells from the second-passage were used for the experiment (Fig 1A).

### Isolation of adipose tissue derived stromal vascular fraction (SVF)

Aseptically, a midline suprapubic skin incision was made to access the bilateral inguinal fat pad of donor rabbits and approximately 6 to 8 g of subcutaneous adipose tissue was obtained from each donor. Then, stromal vascular fraction was isolated as described by Zuk et al. (18). Briefly, adipose tissue was finely minced and washed with phosphatebuffered saline (PBS) and centrifuged at 1200 g for 2 minutes to remove erythrocytes and cellular debris. Samples were then digested in a water bath for 60 minutes at 37˚C by 0.1% collagenase type II (C6885, Sigma-Aldrich, USA) in PBS. After digestion, the collagenase was neutralized by adding an equal volume of DMEM (Sigma-Aldrich, USA). The digestate was centrifuged for 10 minutes at 1200 g at room temperature to separate the SVF from the adipocytes, cellular debris and undigested tissue. After removal of the supernatant that contained mature adipocytes, the cell suspension was filtered through a sterile 100 μm nylon cell strainer into a new tube and centrifuged again. The resulting SVF pellet was re-suspended in PBS and freshly transferred to the operating room for the transplantation procedure (Fig 1B).

### Surgical procedure

**Fig 2:**
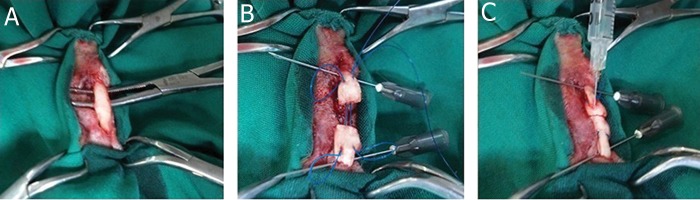
Intraoperative photographs illustrate: A. exposed deep digital flexor tendon, B. modified Kessler suture placement after complete transection of the tendon and C. intratendinous injection of either stromal cells or phosphate-buffered saline (PBS) in tendon stumps.

The model animals were anesthetized using the same anesthetic protocol (see above). One hind limb of each rabbit was randomly prepared for surgical procedure. Skin was incised longitudinally on the plantar aspect of the middle third of the metatarsus over the flexor tendons. The subcutaneous tissues were dissected and the deep digital flexor tendon was exposed. The injury model was a sharp complete transection through the central one third of the tendon. Subsequently, the tendon stumps were sutured with 3/0 monofilament nylon (Ethilon, Ethicon, Inc., USA) in a modified Kessler pattern. Then, 0.2 mL PBS solution that contained 4×10^6^ cultured BMSCs or nucleated cells of freshly isolated SVF was injected intratendinously at the suture site in the treatment group. Control rabbits underwent the identical procedure except that they only received the same volume of PBS solution (Fig 2). The skin was closed with a simple interrupted 3/0 nylon suture. A below stifle plaster cast was applied after surgery and immobilization was continued for two weeks. No antibiotics were used during study period. Three and eight weeks after surgery all rabbits were sacrificed by a thiopental sodium overdose (50 mg/kg, IV, Sandoz, Austria) and the surgical incisions were reopened. Tendons were harvested by proximal and distal transverse incisions approximately 2 cm away from the repair site. Operated tendons were harvested from all animals. For mechanical evaluations, the tendons from both hind limbs of the animals were harvested, wrapped in PBS soaked gauze and immediately stored at -20˚C.

### Mechanical evaluations

Prior to mechanical testing, suture materials were removed and tendons were allowed to thaw while moistened in PBS soaked gauze for 2 hours at room temperature. They were also kept moist by dripping PBS during mounting and mechanical testing. All tendons from the studied groups were submitted to the mechanical test of traction using the H10KS (Hounsfield Ltd., Salfords, UK) testing machine. In order to prevent tendon slippage during tensile testing, 360 grit sandpaper was attached to the ends of each specimen for better clamping. The upper clamp was attached to a 500 N load cell and its displacement was controlled with the aid of a computer endowed with QMat software (version. 2.22, Hounsfield Ltd., Salfords, UK) that was responsible for commanding the equipment and for plotting the force-elongation curve.

The tendons were secured in the clamps and gauge length was defined as the length of the tendon under a 0.5 N pre-load. Under this load, width and thickness of the tendons were measured using a vernier caliper and the cross-sectional area (CSA) was calculated by assuming it to be elliptical.

The dynamic testing took place under axial tension with a constant speed of 50 mm/minute. The mechanical testing consisted of a single-cycle load-to-failure. The force and elongation of the tendon were continuously recorded until the flexor tendon failed. The mode of failure was visually observed and recorded. For each tendon the forceelongation curve was plotted and the following mechanical parameters were obtained: ultimate load (N), yield point (N), stiffness (N/mm), ultimate stress (N/mm^2^), ultimate strain (%), and energy absorption (N.mm).

The ultimate load was defined as the maximum force measured in the tendon during the failure test. The yield point was defined as the point where the curve first deviated from the linear region. Energy absorption values were measured by calculating the area under the force-elongation curve up to the point of maximum force. Ultimate tensile stress was calculated by dividing maximum force values by the initial CSA. Similarly, ultimate tensile strain was calculated by dividing the elongation at the point of maximum force by the initial length. This value was expressed as a percentage. Stiffness was determined as the maximum gradient in the linear region of the force-elongation curve.

### Statistical analysis

Statistical analyses of quantitative results were carried out using PASW Statistics. The residuals were tested for normality by Shapiro-Wilk’s test and normality plots (histograms and quantile plots) and for homogeneity of variation by Levene’s test and examining the residual plot. Normality and/ or homogeneity of variance assumptions for other variables were not satisfied and prior to statistical analysis these variables were logarithmically transformed to fulfill model assumptions. Statistical analysis of data was assessed using one-way analysis of variance (ANOVA). The results are presented as mean and standard deviation (mean ± SD). Multiple comparisons were made by using post-hoc tests (Tukey’s method) to determine which groups significantly differed from each other. Significance was accepted at a family error rate of 0.05.

### Ethical considerations

All protocols were reviewed and approved by the Urmia University’s Ethics Committee before animal experimentation. The maintenance and care of animals complies with Urmia University guidelines for the humane use of laboratory animals.

## Results

No evidence of faulty union and local or systemic complications was observed. Dehiscence of the suture with gap formation between the tendon stumps was not seen in any of the tendons. In addition, there was no noticeable adhesion formation between the tendons and their surrounding tissues in all groups. Failure mode was not influenced by treatment as it was ruptured at the repair site in all tendons.

We observed significant increases in ultimate and yield load, energy absorption, and stress at both time points when treatment groups were compared to their matched controls (p<0.05, Figes3-6).

**Fig 3:**
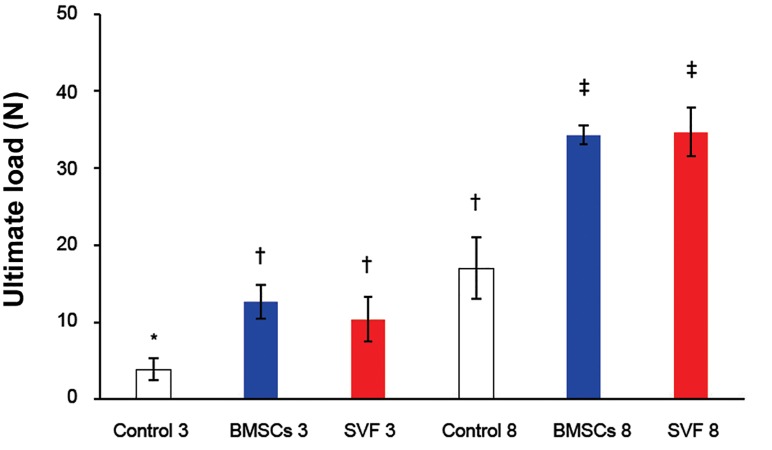
The load values of repairs at the maximum load point at three and eight weeks after surgery. Column heights and error bars represent the group mean and SD. *, †, ‡; Different symbols indicate significant differences among the groups (p<0.05).

**Fig 4:**
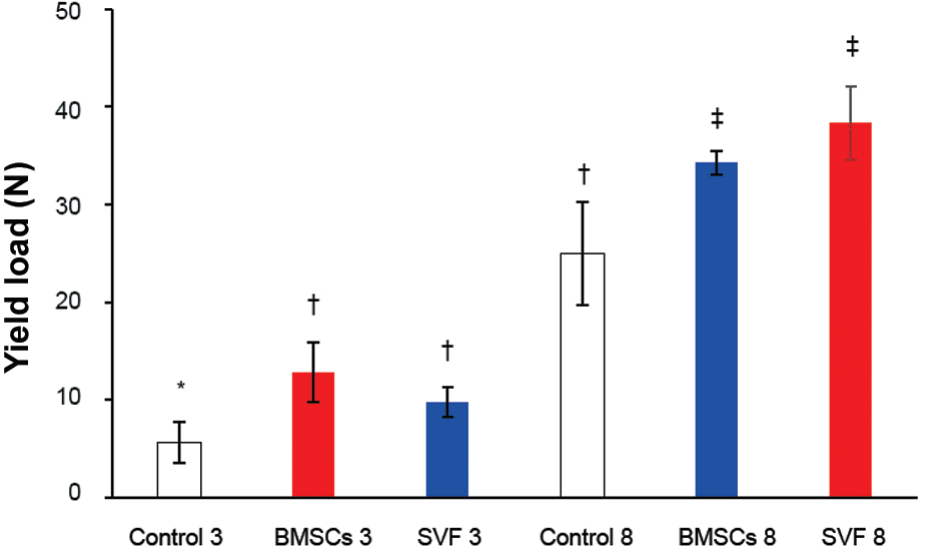
The load values of repairs at the yield point at three and eight weeks after surgery. Column heights and error bars represent the group mean and SD. *, †, ‡; Different symbols indicate significant differences among the groups (p<0.05).

**Fig 5:**
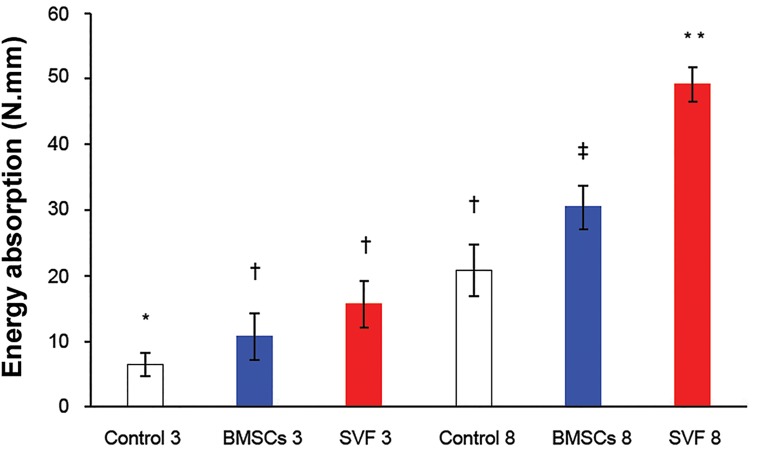
The energy absorption capacity of repairs up to the maximum load point at three and eight weeks after surgery. Column heights and error bars represent the group mean and SD. *, †, ‡; Different symbols indicate significant differences among the groups (p<0.05).

**Fig 6:**
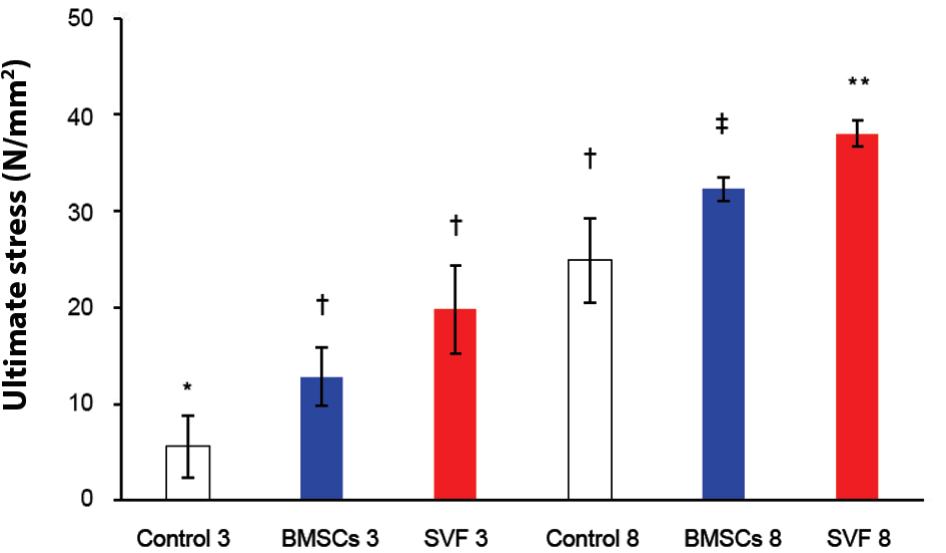
The ultimate stress of repairs at three and eight weeks after surgery. Column heights and error bars represent the group mean and SD. *, †, ‡; Different symbols indicate significant differences among the groups (p<0.05).

**Fig 7:**
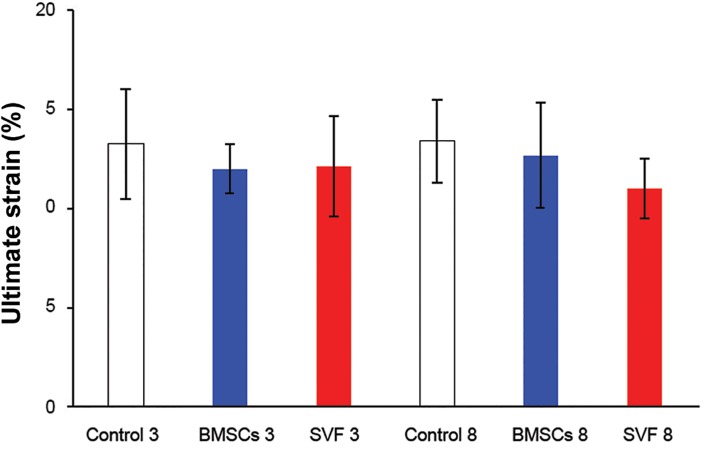
The ultimate strain of repairs at three and eight weeks after surgery. No statistically significant differences were found among the groups (p>0.05). Column heights and error bars represent the group mean and SD.

**Fig 8:**
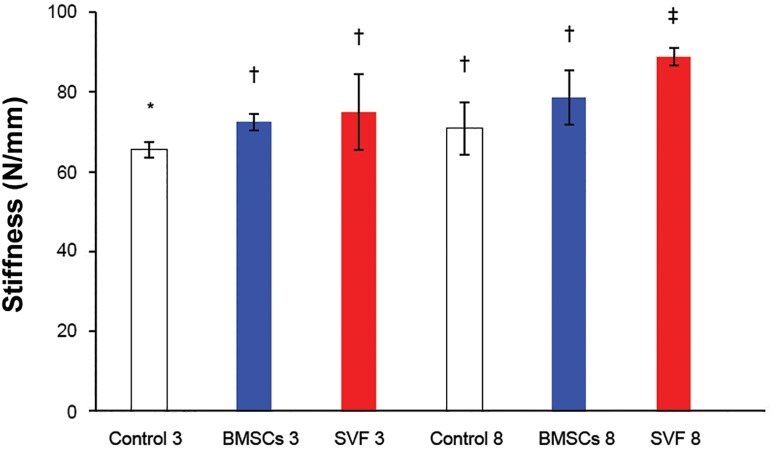
The stiffness of repairs at three and eight weeks after surgery. Column heights and error bars represent the group mean and SD. *, †, ‡; Different symbols indicate significant differences among the groups (p<0.05).

By contrast, there were no statistically significant differences among treatments and their controls either at three or eight weeks after surgery (p>0.05, Fig 7). The lowest value for stiffness was observed in the third week control group, whereas the highest value was observed in the eighth week SVF treated group. There was no significant difference noted among the other groups (Fig 8).

There were no statistically significant differences found when comparing values of all the parameters at the third week between both treatment groups (p>0.05), however, at the eighth week the SVF-treated tendons showed higher degrees of energy absorption, stress and stiffness compared to the BM-treated group (p<0.05).

Time course analysis of results revealed a statistically significant trend of increase in ultimate and yield loads, stress, and energy absorption values within the control and treatment groups from the third to eighth week (p<0.05). Interestingly, values of the all mentioned parameters in BM- and SVF-treated tendons at the third week were not statistically significant when compared to those of controls at eight weeks after surgery, (p>0.05).

## Discussion

Recent improvements in cell therapy using multipotent cells to treat tendon injuries have been exciting and fast forwarding (19). Bone marrow is known as the traditional source of mesenchymal stem cells (20). Several studies have repeatedly demonstrated that transplantation of BMSCs can improve mechanical properties of tendon repairs (16, 17, 21-23). However, collecting bone marrow is still an invasive method with many complications such as infection, bleeding, and chronic pain, therefore limiting its use in tissue engineering and cell therapy (24). On the other hand, collection of stem cells from bone marrow yields only relatively small quantities of viable cells (25).

Adipose derived mesenchymal cells are considered to be a desirable substitute to BMSCs because of their high cell yield and excellent expansion and proliferation abilities (26). Adipose tissue, in contrast, is not in short supply. Per gram, adipose tissue yields a 500-fold greater number of MSCs than bone marrow (27). One of the significant practical factors supporting the therapeutic use of ASCs is the potential to readily prepare these cells for injection within the timeframe of 1-2 hours. Depending on the method of cell isolation and harvesting, approximately 105-106 ASCs per gram of tissue can be obtained, and if required, these cells are easily and rapidly expanded (28).

Several investigations using freshly isolated SVF from adipose tissue in different tissues have reported promising outcomes (29-36), however there are still limited *in vivo* experimental studies that compare the regenerative potential of SVF with those of BMSCs, specifically on tendon repair. Reportedly, among the commonly used evaluation methods for tendon repair, mechanical testing has been considered as the "gold standard" to evaluate efficacy of treatments (37, 38) and previous studies have also suggested that mechanical properties of tendons provide an indication of not only the functional capability of neotendon, but also the recovery level of the tissue material (39, 40). This study has been conducted to compare potential effects of BMSCs and SVF transplantation on mechanical properties of tendon repair after an experimental acute injury

In mechanical testing, the ultimate load indicates maximum tensile load that the material can with stand (41). Our study has shown higher ultimate load and yield load (the amount of tension that causes the sample to break or fail) compared to corresponding controls at three and eight weeks after cell transplantation. To justify any dimensional differences among the specimens, tendon load is reduced to stress by normalization to the tendon CSA (42). The repairs in cell-treated groups have developed significantly greater maximum stress values at both time points. It is believed that significantly higher yield load and stress can be related to collagen organization (16, 43) which is essential to withstand large forces and maximize tensile strength of tendons (44). Repaired tendons should own a great energy-absorbing capacity to store and release high loads to eliminate any damage. According to Witvrouw et al. (45), if this capacity is insufficient, the demands in energy absorption and release may rapidly exceed the tendon capacity and may cause increased risk for re-injury. Thus, increasing the energy capacity of tendons must be one of the key points in the prevention and treatment of tendon injuries. This study has revealed that cell therapy using either source results in significant increase in energy absorption capacity compared to controls in which energy absorption value of the SVF treated group at week eight was higher than the BMSCs treated group. Reportedly, the maximum strain of most tendons is about 8-10% (46). In this study, the value of ultimate strain in SVF-treated group at eighth week has been found to approximate normal levels, which may suggest improved elastic properties compared to the other groups.

According to this study, SVF resulted in significantly higher ultimate stress and energy absorption capacity and stiffness in repairs at the eighth week after transplantations compared to BMSCs treated tendons. We believe higher proliferation rate and longer survival of adipose stromal cells in comparison with BMSC (47, 48) might be the best explanation for these findings. For clinical practice, an ideal approach would be to harvest MSCs and immediately give them back to the patient within the same operation, the so called "onestep surgical procedure" (49). Given the requirements and potential contaminations associated with ex vivo cellular expansion, a simpler procedure would be the use of freshly derived adipose tissue cells for therapy (50). It is claimed that regulatory authorities such as the FDA allow autologous minimally manipulated cell therapy when the procedures do not appreciably change the cells such as differentiation (8).

## Conclusion

The results of the present study showed the efficacy of using uncultured SVF as an alternative to BMSCs in treating tendon repair as its transplantation resulted in increases in most load related properties of tendon repairs.
